# A Case of a 40‐year‐old Female with Speech Disturbance

**DOI:** 10.1002/acn3.70523

**Published:** 2026-09-13

**Authors:** Victoria Lorah, Michael V. Robers

**Affiliations:** ^1^ Department of Neurology Barrow Neurological Institute at Phoenix Children’s Phoenix Arizona USA; ^2^ Department of Neurology Barrow Neurological Institute Phoenix Arizona USA

**Keywords:** Baló's concentric sclerosis, demyelination, tumefactive lesions

## 1 Summary of Case (HPI, Relevant Exam Findings, and Relevant Data)

A 40‐year‐old Caucasian female with a history of migraines and depression presented to the emergency department for word‐finding difficulty, speech disturbances, and “feeling off” for two days. Her husband reported that it sounded like she had a lisp when she spoke. She endorsed brain fog but denied fever, chills, headache, dysuria, focal weakness, or numbness/tingling. She denied a social history of smoking, alcohol, or drug use. She was otherwise healthy with no other medical or surgical history, including no history of stroke, cardiovascular disease, or other neurologic issues outside of migraines. Imaging showed multiple enhancing white matter lesions consistent with demyelination. An initial diagnosis of multiple sclerosis was given, and she completed 5 days of high‐dose steroids. The patient would return to the hospital within a few days of discharge after developing left‐sided weakness. Over the next month, the patient's demyelinating lesions would become tumefactive, with characteristic concentric rings seen in Baló's concentric sclerosis. She had oligoclonal bands in her CSF, and the brain biopsy was consistent with a demyelinating lesion, but otherwise her work‐up was unremarkable. Despite treatment with steroids, plasmapheresis, immunoglobulins, and cyclophosphamide, the patient's neurologic status rapidly declined, and she succumbed to sepsis (Figures 1, 2, 3, 4, 5).

**FIGURE 1:**
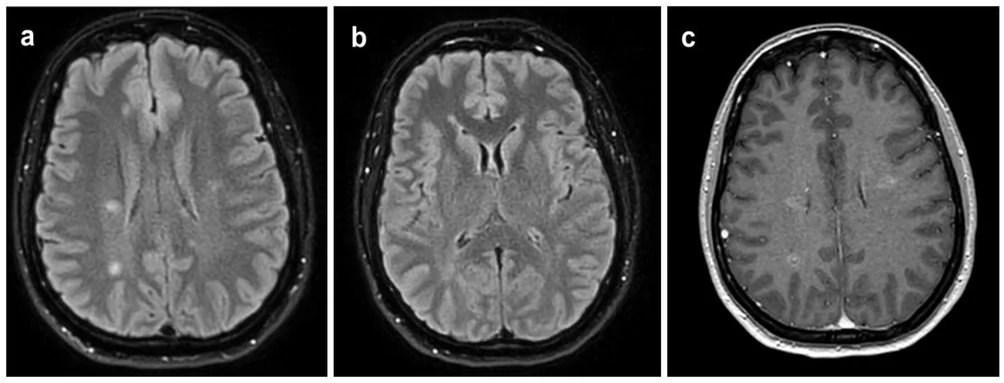
MRI brain axial FLAIR view obtained on day 4 after symptom onset, demonstrating multiple enhancing and non‐enhancing white matter lesions in the periventricular area (images a and b). Post‐contrast image included (image c). This was the patient's first MRI.

**FIGURE 2:**
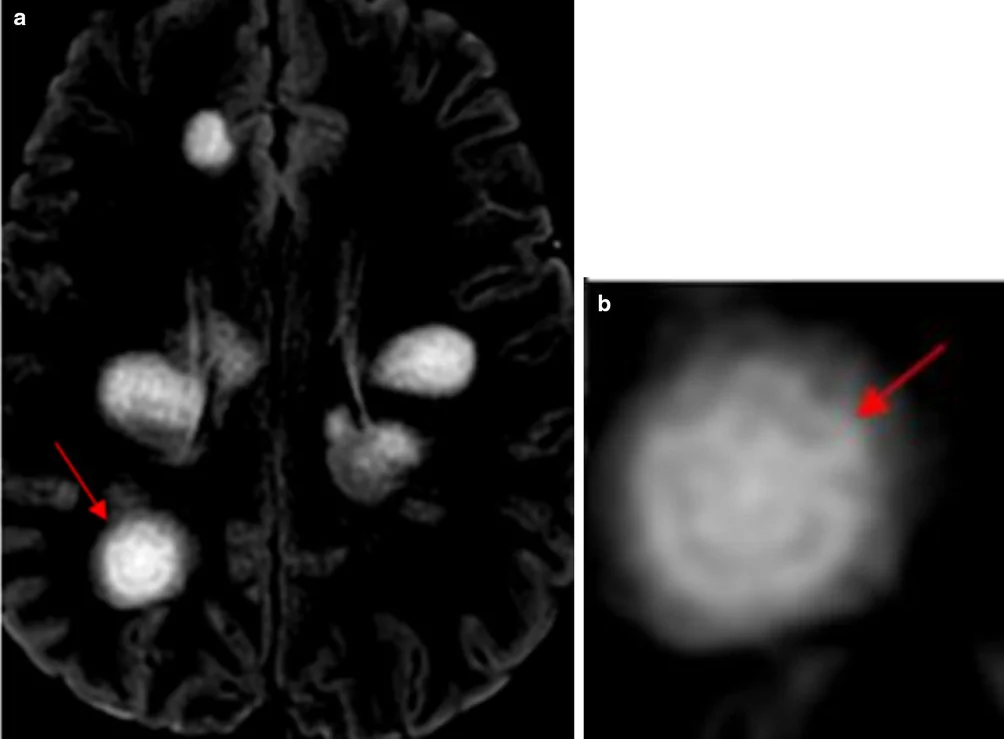
MRI Brain axial FLAIR view obtained on day 18 after symptom onset, showing multiple white matter lesions, with arrows denoting concentric layers of demyelination (a). Concentric layers are better seen with the magnified image (b).

**FIGURE 3:**
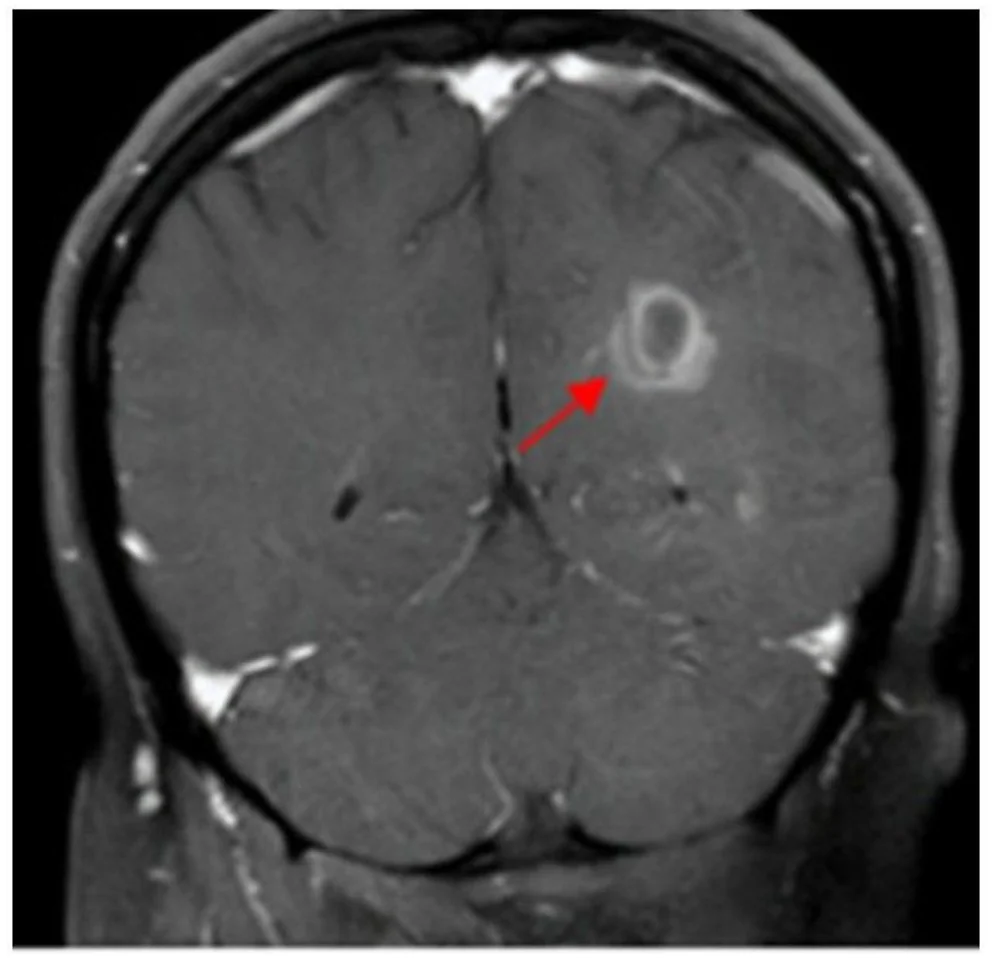
Post‐contrast MRI Brain coronal view obtained day 18 after symptom onset, which demonstrates a full ring of layered enhancement, in addition to a separate partial ring of enhancement around the same lesion (arrow).

**FIGURE 4:**
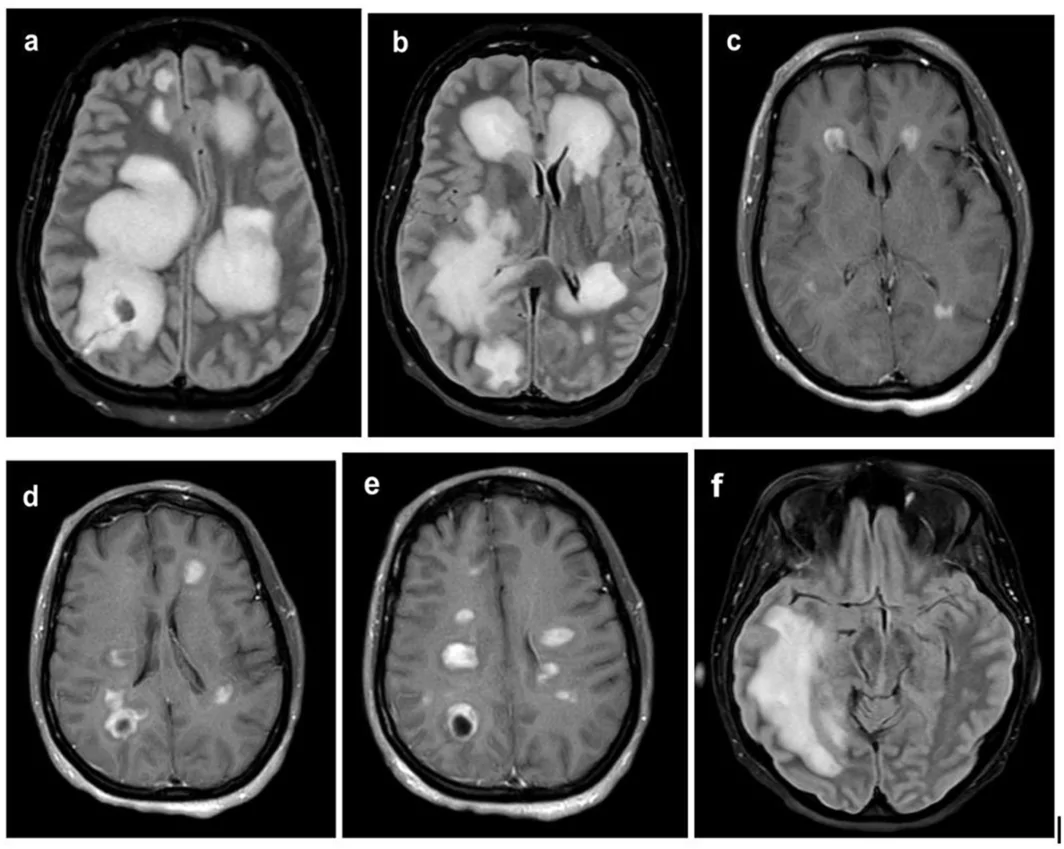
MRI brain Axial FLAIR slices obtained on day 43 of symptom onset, demonstrating significantly increased edema surrounding diffuse concentric demyelinating white matter lesions with leftward midline shift (a–c). These images were obtained after the completion of high‐dose intravenous steroids, plasma exchange, intravenous immunoglobulin, and cyclophosphamide therapies. Post‐contrast axial T1 views within the same study (d–f).

**FIGURE 5:**
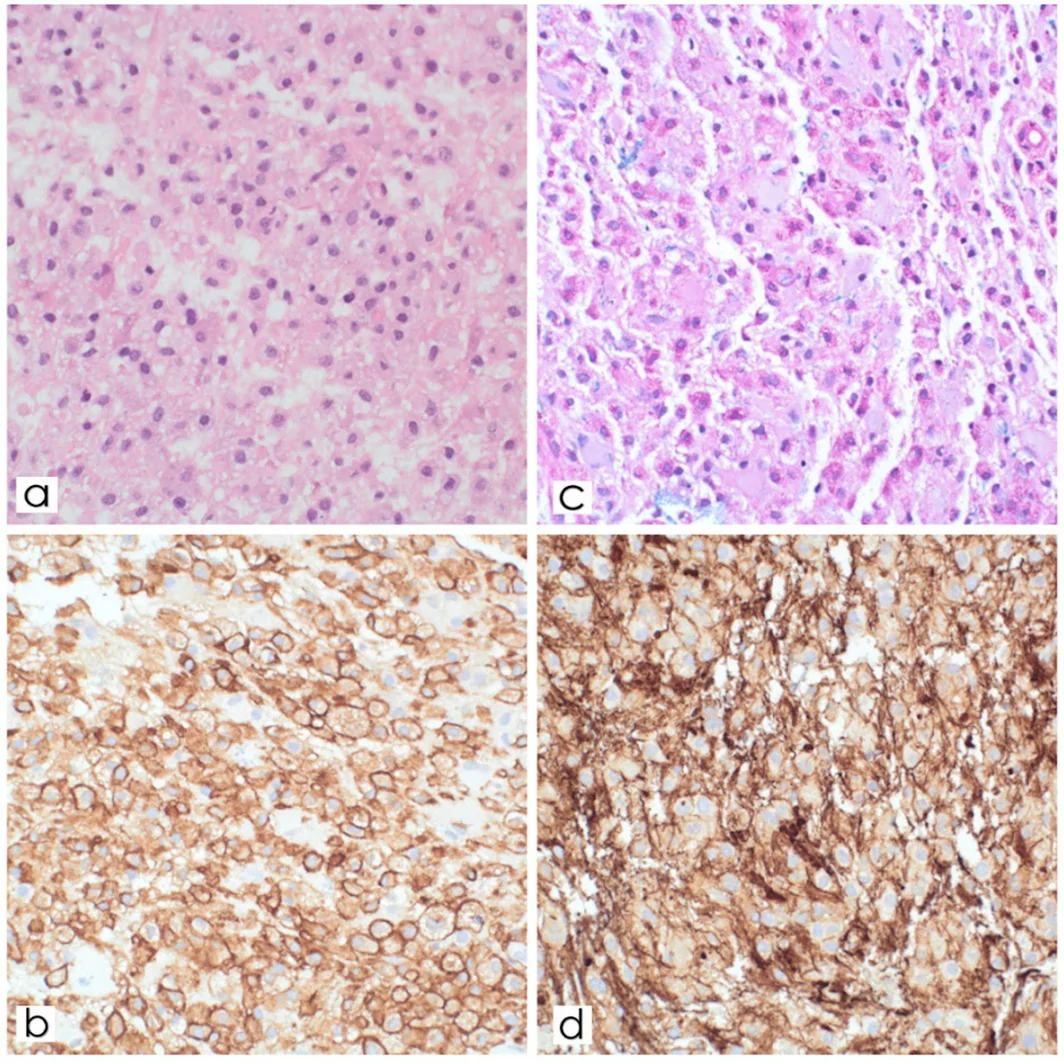
Tissues acquired from brain biopsy of the demyelinating concentric lesion. Hematoxylin and eosin‐stained sections (a) show sheets of macrophages, which are highlighted with a CD163 immunostain (b). LFB‐PAS stain shows loss of myelination and some PAS‐positive material within macrophages (c). Neurofilament shows relative axonal preservation (d).

## 2 Diagnosis

Baló's concentric sclerosis [1].

## 3 Take‐Home Points

- Demyelinating lesions have a wide range of clinical presentations, but Balo's concentric sclerosis will typically present with rapid and severe progression compared to standard multiple sclerosis [1].
- Balo's concentric sclerosis has a distinct “onion ring” appearance on imaging and oligodendrocytopathy pattern (also known as pattern III) on histology [2].
- Treatment of Balo's concentric sclerosis is similar to multiple sclerosis, including high‐dose intravenous steroids, plasmapheresis, and immunoglobulin. The difference is Balo's concentric sclerosis is more likely to require aggressive treatment with multiple rounds of these medications and addition of disease‐modifying therapy [3].

## Funding

The authors have nothing to report.

## Conflicts of Interest

The authors declare no conflicts of interest.

## Data Availability

Data sharing not applicable to this article as no datasets were generated or analysed during the current study.

## References

[1] S. Jarius , C. Würthwein , J. R. Behrens , et al., “Baló's Concentric Sclerosis is Immunologically Distinct from Multiple Sclerosis: Results from Retrospective Analysis of Almost 150 Lumbar Punctures,” Journal of Neuroinflammation 15 (2018): 22, 10.1186/s12974-017-1043-y. PMC5774135 29347989

[2] T. A. Hardy and D. H. Miller , “Baló's Concentric Sclerosis,” Lancet Neurology 13, no. 7 (2014): 740–746, 10.1016/s1474-4422(14)70052-3. 24943346

[3] E. A. Jolliffe , Y. Guo , T. A. Hardy , et al., “Clinical and Radiologic Features, Pathology, and Treatment of Baló Concentric Sclerosis,” Neurology 97, no. 4 (2021): e414–e422, 10.1212/WNL.0000000000012230. PMC8362356 34011576

